# Anthrax and Gulf War Illness (GWI): Evidence for the Presence of Harmful Anthrax Antigen PA63 In the Serum of Veterans with GWI

**DOI:** 10.29245/2572.942x/2019/6.1255

**Published:** 2019-11-25

**Authors:** Effie-Photini C. Tsilibary, Eric P. Souto, Marian Kratzke, Lisa M. James, Brian E. Engdahl, Apostolos P. Georgopoulos

**Affiliations:** 1 Brain Sciences Center, Department of Veterans Affairs Health Care System, Minneapolis, Minnesota, USA; 2 Department of Neuroscience, University of Minnesota Medical School, Minneapolis, Minnesota, USA; 3 Department of Psychiatry, University of Minnesota Medical School, Minneapolis, Minnesota, USA; 4 Department of Psychology, University of Minnesota, Minneapolis, Minnesota, USA; 5 Department of Neurology, University of Minnesota Medical School, Minneapolis, Minnesota, USA

**Keywords:** Gulf War Illness, anthrax vaccine, neuroblastoma culture, PA63

## Abstract

Gulf War Illness (GWI) is a multisystem disorder of unknown etiology that has afflicted many veterans of the 1990-91 Gulf War who have sustained progressively worsening health since the war^1^. Recent studies have demonstrated the presence of active inflammation in GWI^2,3^ and, in addition, a positive association of the levels of C-reactive protein (CRP), an inflammatory marker, with GWI symptom severity^3^. Moreover, we have shown that GWI serum contains substances that are harmful to neural cultures^4^`, a detrimental effect that can be prevented by serum of healthy GW veterans^4^ and partially so by pooled human immunoglobulin G (IgG)^5^. Although possible exposure to environmental toxins in war theater has been traditionally blamed for GWI^6^, the evidence above^3-5^ and the fact that the disease also afflicted nondeployed veterans^7^, point to other causes, including the vaccines administered to GW veterans^4,5,7^, such as the vaccine against anthrax. Here we present, for the first time, evidence indicating the presence of the harmful anthrax protective antigen PA63 in the serum of 15 veterans suffering from GWI, as follows. First, we confirmed that the addition of GWI serum to the culture had a detrimental effect, including decreased cell spreading and increased cell apoptosis, as reported previously^4^. And second, we found that the concomitant addition of specific polyclonal or monoclonal antibodies against PA63 had a remarkable protective effect on N2A cultures, significantly ameliorating cell spreading and reducing cell apoptosis. These results document that the adverse effects of GWI serum on neural cultures are due, in part, to persistent pathogens derived from the anthrax vaccine. We hypothesize that these anthrax pathogens persisted in the blood of the GWI veterans tested because of inability of those veterans to make antibodies against them, probably due to lack of Human Leukocyte Antigen (HLA) protection^8^. Finally, our findings point to a possible successful intervention in GWI consisting in neutralizing (by administering specific antibodies) and/or removing (by plasmapheresis) those harmful anthrax antigens.

## Introduction

After the Persian Gulf War of 1990-91, about one third (>200,000) deployed veterans complained of a variety of chronic physical and neurocognitive symptoms^1,9-11^, which are presently identified as Gulf War Illness (GWI). We previously described a number of functional and structural brain abnormalities in GWI, including changes in synchronous neural communication patterns^12-17^ and the presence of subcortical brain atrophy in certain GWI patients^13^. This atrophy was absent in veterans carrying the Human Leukocyte Antigen (HLA) allele DRB1*13:02^15^, one of six HLA class II alleles that we had reported previously as protective for GWI, in a dose-response fashion^8^. The function of HLA class II alleles is specific immunity, namely to match to external antigens and present them to CD4^+^ lymphocytes leading ultimately to the production of specific antibodies by B cells to neutralize the offending antigen^18^. Given these considerations, we hypothesized that the lack of HLA class II protection observed in GWI would have allowed offending antigens to persist^15^.

Indeed, administered antigens have been reported to persist for prolonged times following immunization in lymphatic endothelial cells^19^ and lymphoid follicles of specifically immunized mice^20^. Therefore it is feasible that one or several antigens/pathogens persisted in GWI patients circulating in the blood stream, hence our “persistent antigen” hypothesis^15^. We further hypothesized that such persisting antigens could have come from vaccines to which GW veterans were exposed, thus leading to multi-symptom chronic disease stemming from cell damage, low grade inflammation^2,3^, and possibly other mechanisms. According to this hypothesis, healthy GW veterans carrying protective alleles^8^ would have specific antibodies in their blood, which could neutralize the hypothesized persistent antigens present in GWI serum. As a first step in testing this prediction, (a) we assessed the effect of GWI serum on function and morphology of neural cultures (primary neuronal cells and neuroblastoma 2A (N2A) cells in vitro, and (b) we tested serum from healthy GW veterans^4^. Indeed, we found that (a) GWI serum exerted harmful effects on neural cultures, since it compromised cell-cell communication, cell spreading and cell survival by enhancing cell apoptosis, and (b) those effects were prevented by the addition of serum from healthy GW-era veterans^4^. These findings suggest that healthy serum may contain, among other, antibodies against harmful antigens present in GWI serum; if so such antibodies may hold promise for a successful intervention in treating GWI. As a first step in testing this hypothesis, we assessed the effect of pooled human antibodies in vitro, by adding to the culture pooled human immunoglobulin G (IgG)^5^. Although pooled IgG does not come from GW-era veterans, it should contain antibodies against a broad range of pathogens, partially overlapping with some of those contained in the vaccines administered to GW veterans. However, rare pathogens such as anthrax antigens should not be present in pooled human IgG, the presence of which exerted a partial beneficial effect in N2A cultures^5^.

In this study, we specifically investigated the effects of antibodies against the anthrax protective antigen PA63. This antigen is associated with the anthrax vaccine which was administered to GW-veterans in the form of proteins, mainly PA83, contained in the “Biothrax” vaccine (https://www.rxlist.com/biothrax-drug.htm#indications). In vivo PA83 binds to its receptor and is cleaved by furin family proteases to a 63-kD protein (PA63)^21^. Here we used specific anti-PA63 antibodies co-incubated with GWI serum and observed a pronounced protective effect in N2A cells. These results implicate PA63 as an active, harmful substance present in the serum of GWI veterans and suggest that its neutralization and/or removal from the blood of GWI patients could be a useful intervention for GWI in the future.

## Materials and Methods

### Serum.

We used serum from 15 GWI veterans with substantial GWI symptoms and serum from a healthy GW veteran; patients did not carry any of the 6 GWI-protective alleles^8^, whereas the control carried 2 such alleles. The study was approved by the relevant Institutional Review Board and informed consent for using their serum was obtained from all participants.

### Cell culture.

Neuro-2A neuroblastoma (N2A) cells were cultured in Eagle’s minimal essential medium (EMEM, ATCC, VA, USA) containing 10% fetal bovine serum (ThermoFisher Scientific, Waltham, MA) in poly-D-lysine coated, 24-well plates at a concentration of 30,000-50,000/well for 48-72h. The medium was then changed to Neurobasal containing N2 supplement and L-glutamine (ThermoFisher Scientific, Waltham, MA), in the absence (medium control) or presence of human serum (healthy or GWI). For all experiments, human serum was added in 3 combinations: control (10%), GWI (10%) and GWI preincubated with anthrax antibodies.

### Cell morphology / Process formation assay.

The effect of anthrax antibodies to antigens contained in the GW-era vaccines on the morphology of N2A cells was examined. For this assay, anti-AnthraxProtective Antigen (polyclonal antiserum Cat.No.CPBT-66806RA), and monoclonal anti-anthrax antibody (CABT-51076MA, both from Creative Diagnostics, Shirley, NJ) were used in parallel with similar results. Both antibodies were titrated for effects at a series of concentrations following pre-incubation with GWI serum, and used at the lowest active concentration, (15% for polyclonal anti-anthrax antiserum, and 5μg/ml of monoclonal anti-anthrax). Each antibody was incubated with 100 μl of GWI serum for 60 min at 37°C, and then added in a final volume of 1ml of Neurobasal medium containing N2 supplement and L-glutamine.

The N2A cells were cultured with GWI serum pre-incubated in the presence or absence of each of these antibodies for 2 more days and the cells were photographed. Images were obtained from 5-8 different fields per sample, from a minimum of three experiments using a Motic AE2000-Trinocular inverted microscope (Ted Pella, Redding, Ca), with a Zeiss Axiocam 105 color digital camera (Carl Zeiss Microscopy, LLC, Thornwood, NY). The extent of cell spreading was then calculated with ImageJ software by measuring the number of cells with processes relative to the total cell number.

### Cell Apoptosis with Terminal deoxynucleotidyl transferase mediated dUTP Nick End Labeling assay (TUNEL) assay.

The extent of cell apoptosis of Neuro-2A cells was examined at 2 days post-exposure to GWI serum and GWI pre-incubated with antiPA63 antibody, using 4- and 8-chamber glass slides (ThermoFisher Scientific, Waltham, MA) coated with poly-D-lysine at 50μg/ml as mentioned above. N2A cells were seeded at a concentration of 50,000-100,000 cells per chamber, in 1 ml of Neurobasal/N2/ L-glutamine medium for 2 days. In sequence, 10% of GWI serum, incubated for 60 min at 37°C in the presence or absence of 1 μg of anthrax antibodies or 15% polyclonal anthrax antiserum were added for 2 more days. At the end of the incubation period, the cells were examined for apoptosis. Apoptotic cells were detected using the In Situ Cell Death Detection Kit, TMR red (Terminal deoxynucleotidyl transferase (TdT) enzyme and fluorochrome labeling solution) according to the manufacturer’s protocol. Briefly, the cells were fixed in ice-cold methanol for 10 min at room temperature, rinsed with PBS and permeabilized with 0.1% Triton X-100 in PBS for 3 min on ice. The cells were then incubated with 150μl of TUNEL reaction mixture for 60 min at 37°C in the dark (In situ Cell Death Detection Kit, TMR red, (ThermoFisher scientific, Waltham MA), or Click-iT Alexa Fluor488 Assay (ThermoFisher scientific, Waltham MA). The cells were then washed 3X with PBS and Diamond AntiFade mounting medium with 4’,6-diamidino-2-phenylindole (DAPI) stain (ThermoFisher Scientific, Waltham, MA) was used for visualization of nuclei, using the EVOS FL Cell Imaging System (ThermoFisher Scientific, Waltham, MA). Eight-10 images were obtained from different fields from a minimum of two experiments with each different experimental condition. Apoptosis was then calculated with ImageJ software by measuring the number of TUNEL-labeled cells (red nuclei) relative to the total cell number (DAPI-stained nuclei).

### Data analysis.

A repeated measures analysis of variance (rANOVA) was used to evaluate the effect of anti-anthrax antibody on N2A cell spreading and apoptosis, given that serum each of the 15 GWI patients was applied to N2A cells in the absence and presence of co-incubated antibody, and tested concurrently with serum from the control in quintuplicate. In total, six such rANOVAs were carried out, namely 3 for the spreading (GWI vs. Control, GWI vs. GWI+anti-Anthrax Antibody [aAA], GWI+aAA vs. Control) and the same 3 for apoptosis. The 2 treatments in each pair were the “within subjects” factor. The IBM-SPSS statistical package (version 25) was used for all analyses.

## Results

### Cell spreading: Group comparisons

**Figure 1.**
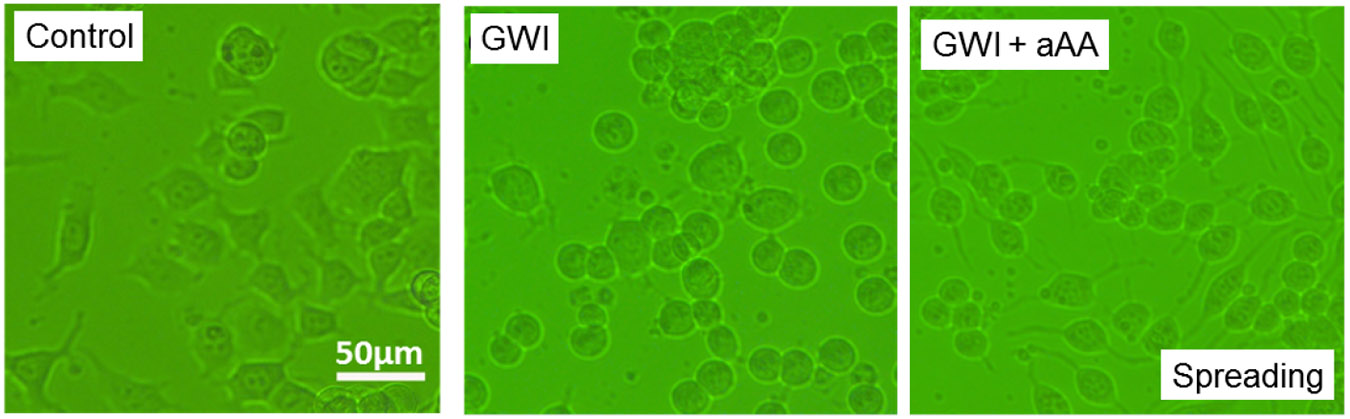
Representative examples of spreading of N2A cultured in the presence of Control and GWI serum, and GWI serum with anti-Anthrax Antibody (aAA). See text for details.

Fig. 1 shows representative images of cell spreading for the GWI, GWI+aAA, and Control treatments. It can be seen (a) that cell spreading was reduced by the addition of GWI serum, relative to the control, and (b) that this negative effect was ameliorated by the addition of anti-Anthrax Antibody (aAA). These effects were quantitatively assessed, with the following results.

#### *GWI vs. Control* (Fig. 2).

Cell spreading was substantially reduced in GWI vs. Control (27.4% vs. 45.6%, respectively). This effect was highly statistically significant (t_[74]_ = 15.48, P = 6.6×10^−25^). The effect size in the rANOVA (eta squared) was 0.764.

#### *GWI vs. GWI+aAA* (Fig. 3).

The addition of anti-Anthax Antibody to the GWI serum improved substantially cell spreading, from 27.4% to 40.9%, respectively). This effect was highly statistically significant (t_[74]_ = 17.31, P = 9.9×10^−28^). The effect size in the rANOVA (eta squared) was 0.802.

#### *GWI+aAA vs. Control* (Fig. 4).

Cell spreading in GWI+aAA, although higher than in GWI, it was still lower than the Control (40.9% vs. 45.6%, respectively). This effect was statistically significant (t_[74]_ = 3.58, P = 0.0006). The effect size in the rANOVA (eta squared) was 0.148.

### Cell apoptosis: Group comparisons

**Figure 5.**
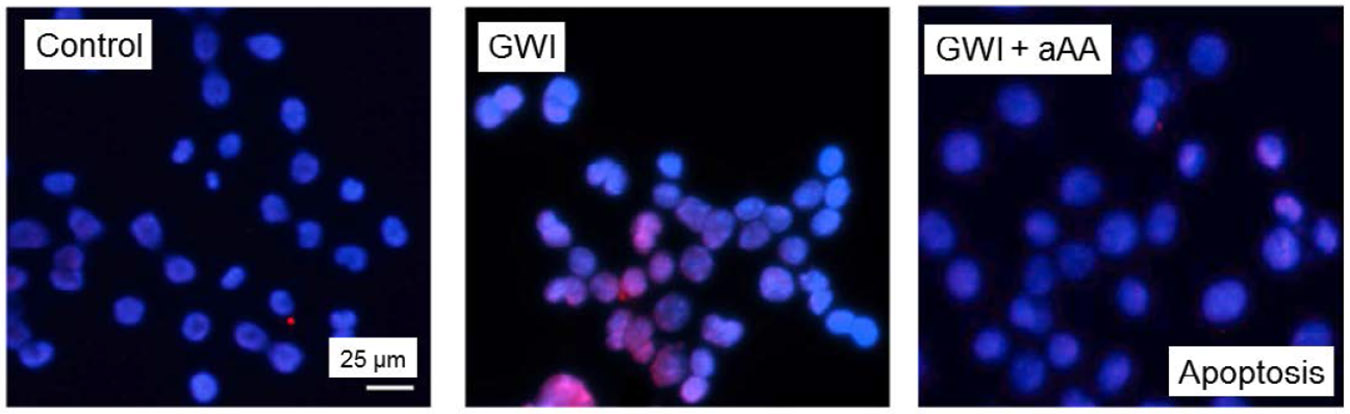
Representative example of cell apoptosis. Nuclei from N2A cells incubated with Control, GWI, and GWI+aAA serum. Healthy nuclei appear blue (DAPI stain); apoptotic nuclei appear red (TUNEL TMR-Red stain). See text for details.

Fig. 5 shows representative images of cell apoptosis for the GWI, GWI+aAA, and Control treatments. It can be seen (a) that cell apoptosis increased by the addition of GWI serum, relative to the control, and (b) that this negative effect was ameliorated by the addition of anti-Anthrax Antibody (aAA). These effects were quantitatively assessed, with the following results.

#### *GWI vs. Control* (Fig. 6).

Cell apoptosis increased substantially in GWI vs. Control (28.5% vs. 7.8%, respectively). This effect was highly statistically significant (t_[74]_ = 19.00, P = 3.4×10^−30^). The effect size in the rANOVA (eta squared) was 0.830.

#### *GWI vs. GWI+aAA* (Fig. 7).

The addition of anti-Anthax Antibody to the GWI serum improved substantially reducing cell apoptosis, from 28.5% to 12.7%, respectively). This effect was highly statistically significant (t_[74]_ = 15.2, P = 3.4×10^−24^). The effect size in the rANOVA (eta squared) was 0.753.

#### *GWI+aAA vs. Control* (Fig. 8).

Cell apoptosis in GWI+aAA, although lower than in GWI, it was still higher than the Control (12.7% vs. 7.8%, respectively). This effect was statistically significant (t_[74]_ = 8.27, P = 4.0×10^−12^). The effect size in the rANOVA (eta squared) was 0.480.

### Consistency of the effects

**Figure 9.**
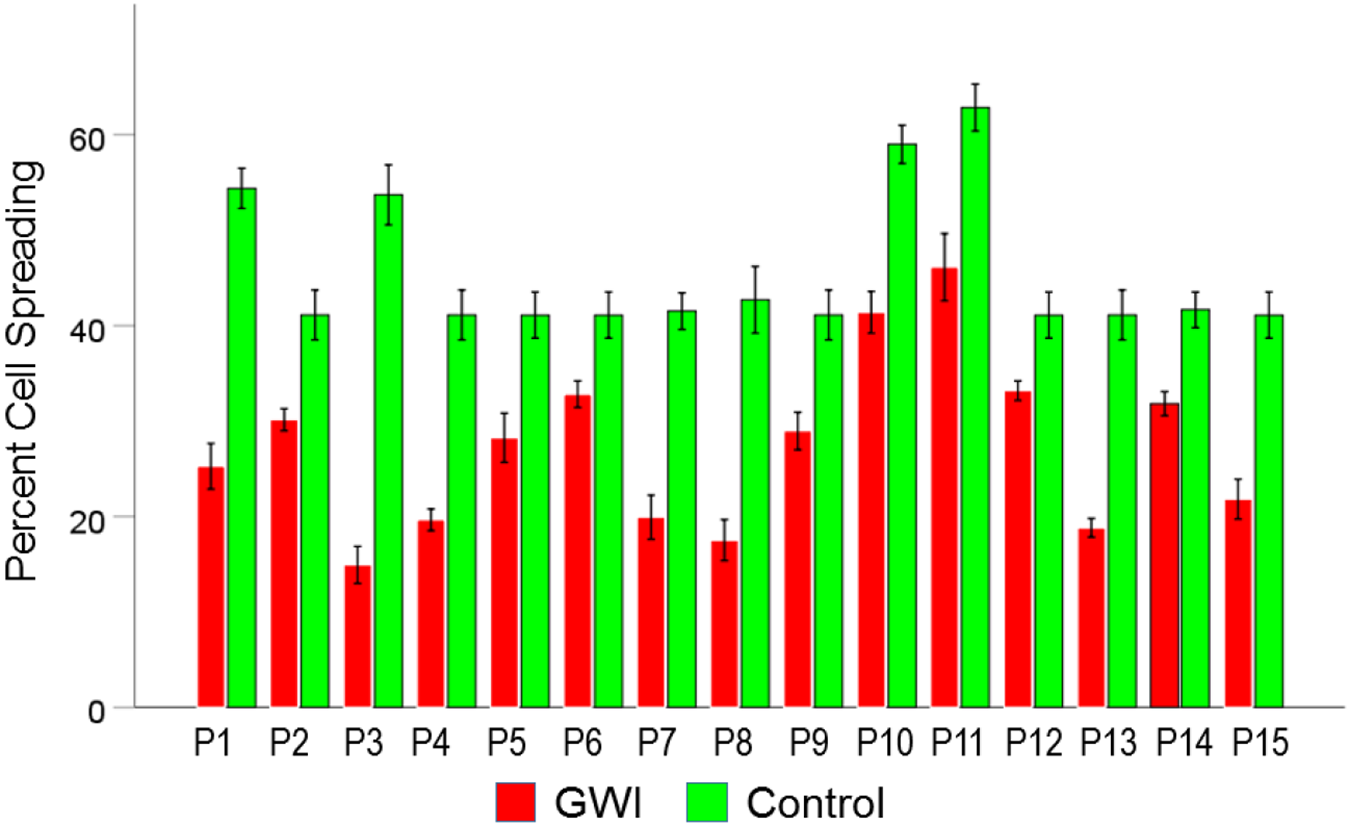
Mean (± SEM) percent cell spreading for each one of the 15 GWI patients (P1-P15, N = 5 per patient) in the conditions shown (GWI, Control). Notice the systematic decrease of cell spreading (relative the Control), present in each patient. See text for details.

**Figure 10.**
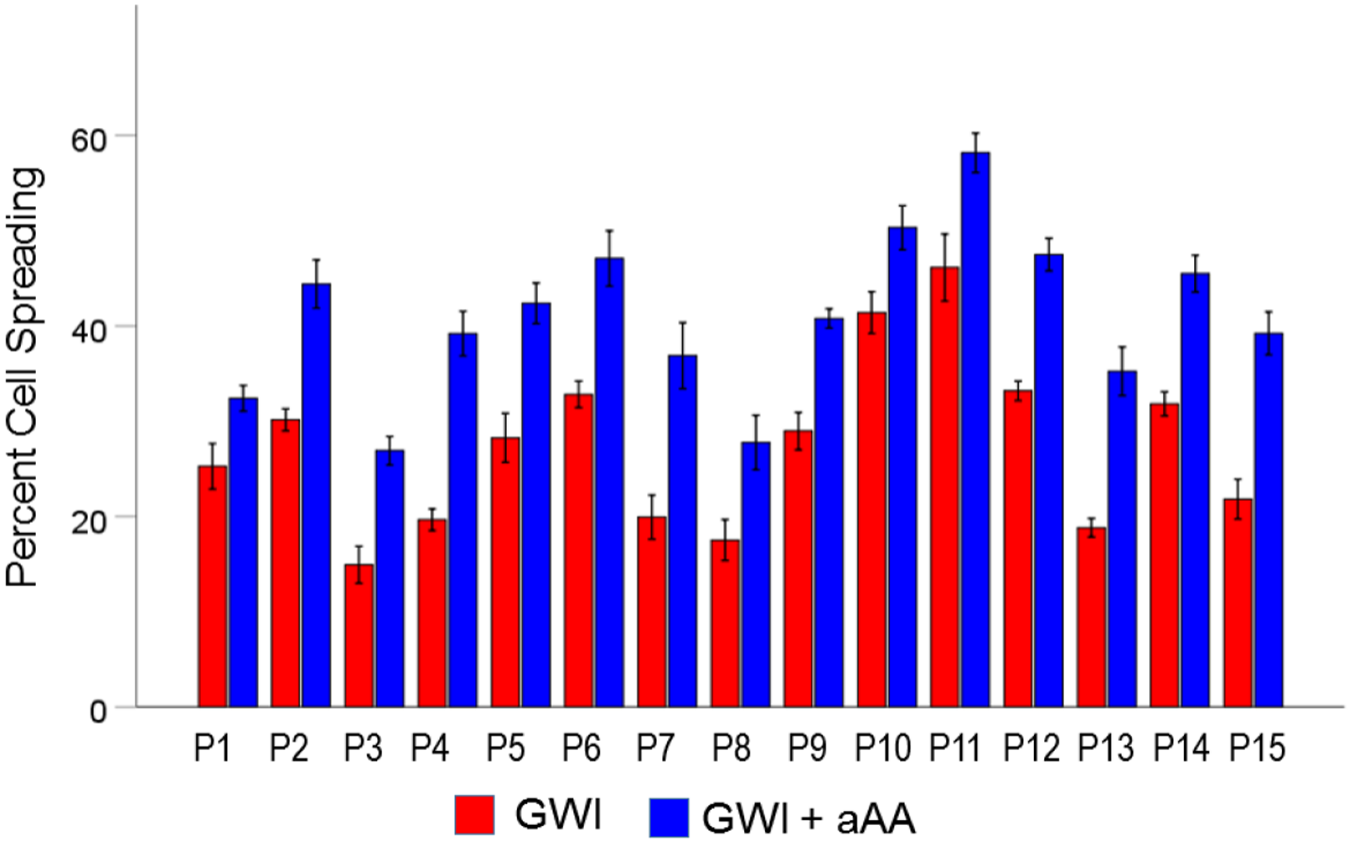
Mean (± SEM) percent cell spreading for each one of the 15 GWI patients (P1-P15, N = 5 per patient) in the conditions shown (GWI, GWI+aAA). Notice the systematic improvement (increase) of cell spreading effected by the addition of anti-Anthrax Antibody, present in each patient. See text for details.

**Figure 11.**
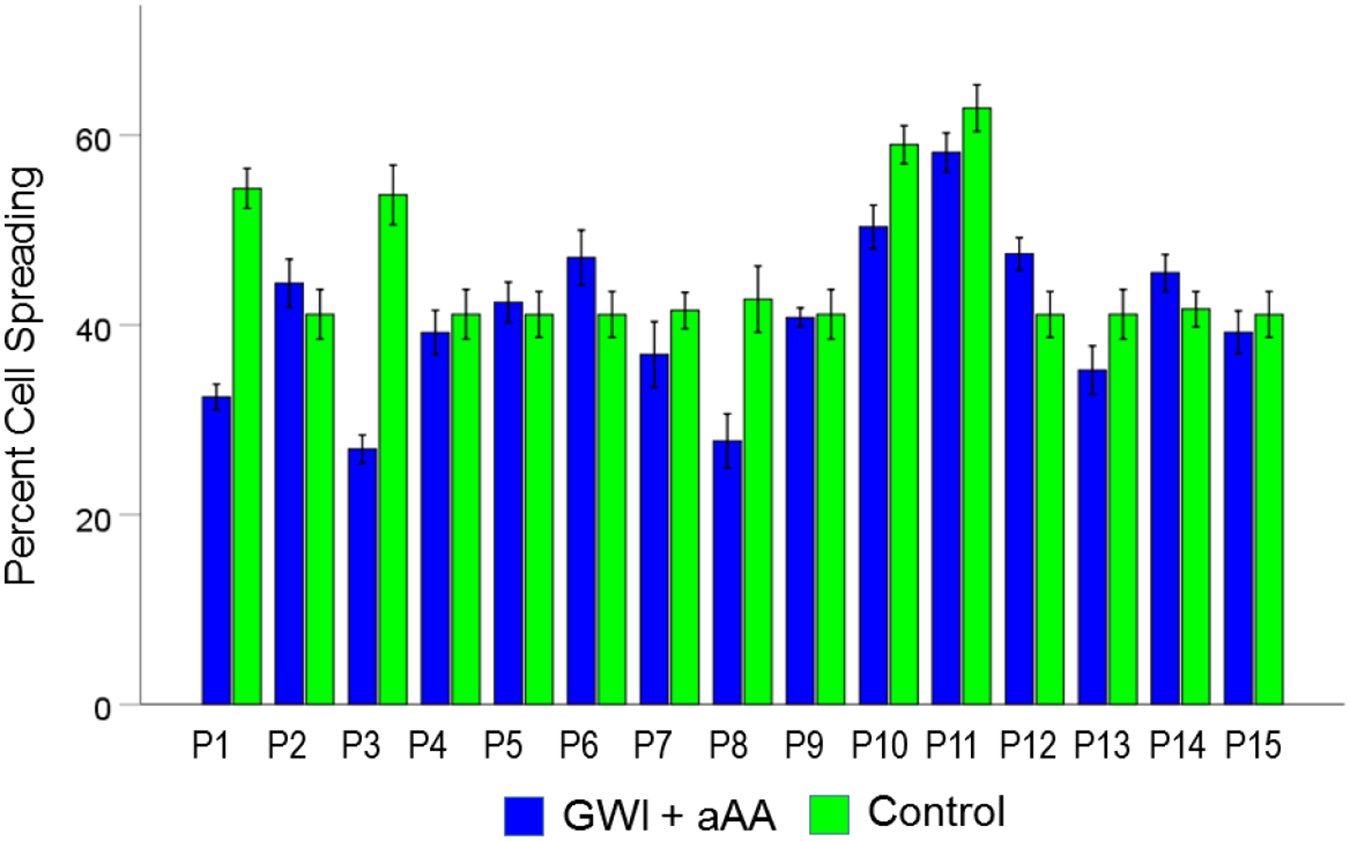
Mean (± SEM) percent cell spreading for each one of the 15 GWI patients (P1-P15, N = 5 per patient) in the conditions shown (GWI+aAA, Control). Notice that cell spreading in the GWI+aAA condition is close but systematically lower than that in Control. See text for details.

**Figure 12.**
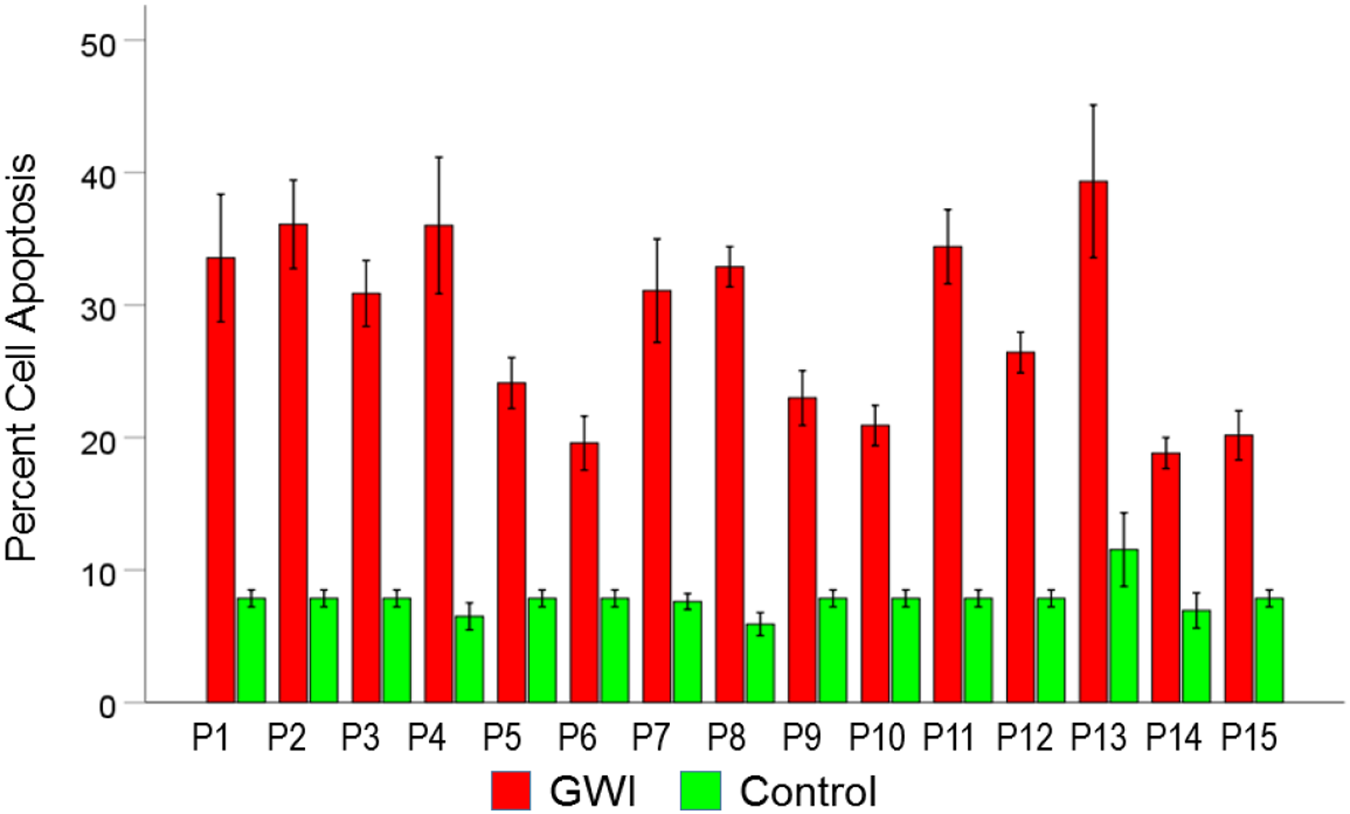
Mean (± SEM) percent cell apoptosis for each one of the 15 GWI patients (P1-P15, N = 5 per patient) in the conditions shown (GWI, Control). Notice the systematic increase of cell apoptosis (relative the Control), present in each patient. See text for details.

**Figure 13.**
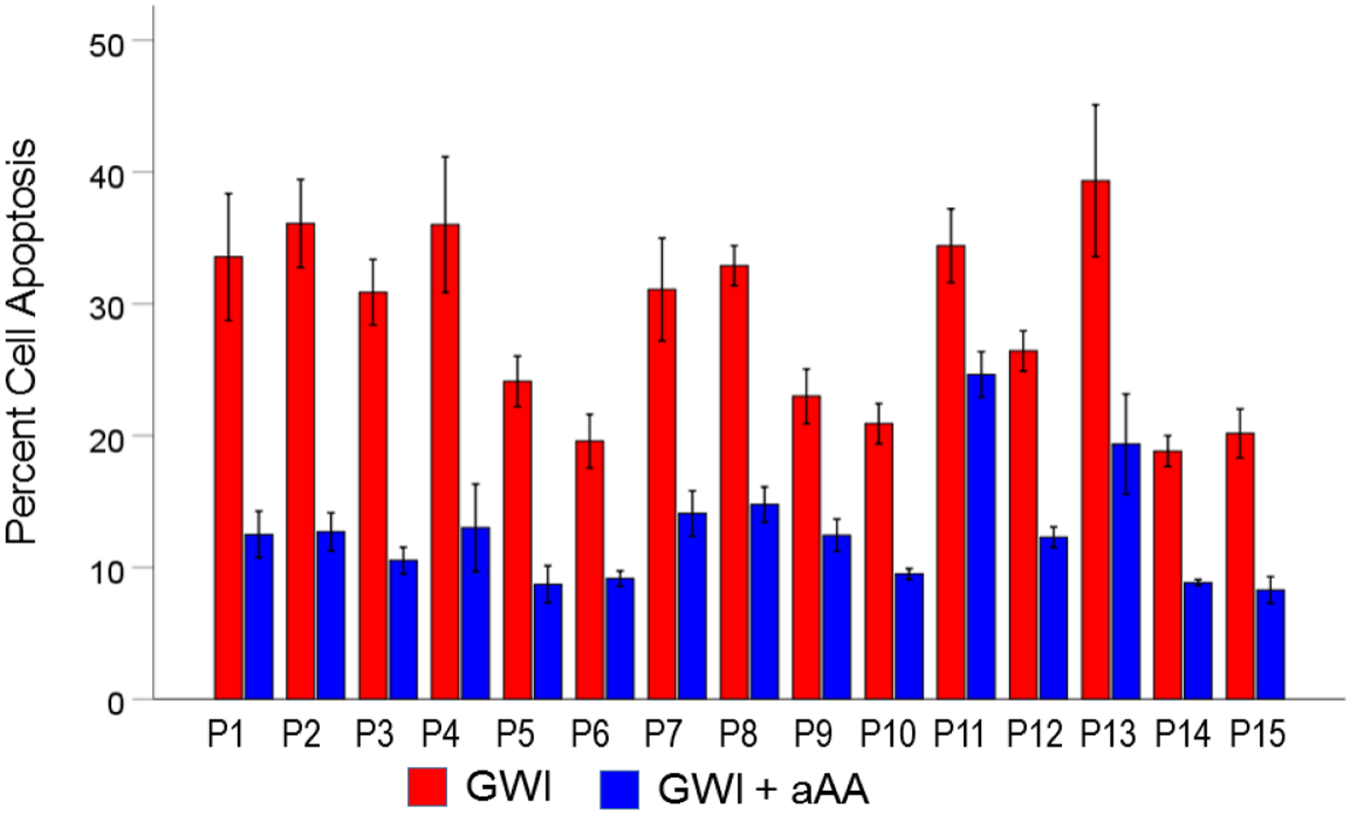
Mean (± SEM) percent cell apoptosis for each one of the 15 GWI patients (P1-P15, N = 5 per patient) in the conditions shown (GWI, GWI+aAA). Notice the systematic improvement (decrease) of cell apoptosis effected by the addition of anti-Anthrax Antibody, present in each patient. See text for details.

**Figure 14.**
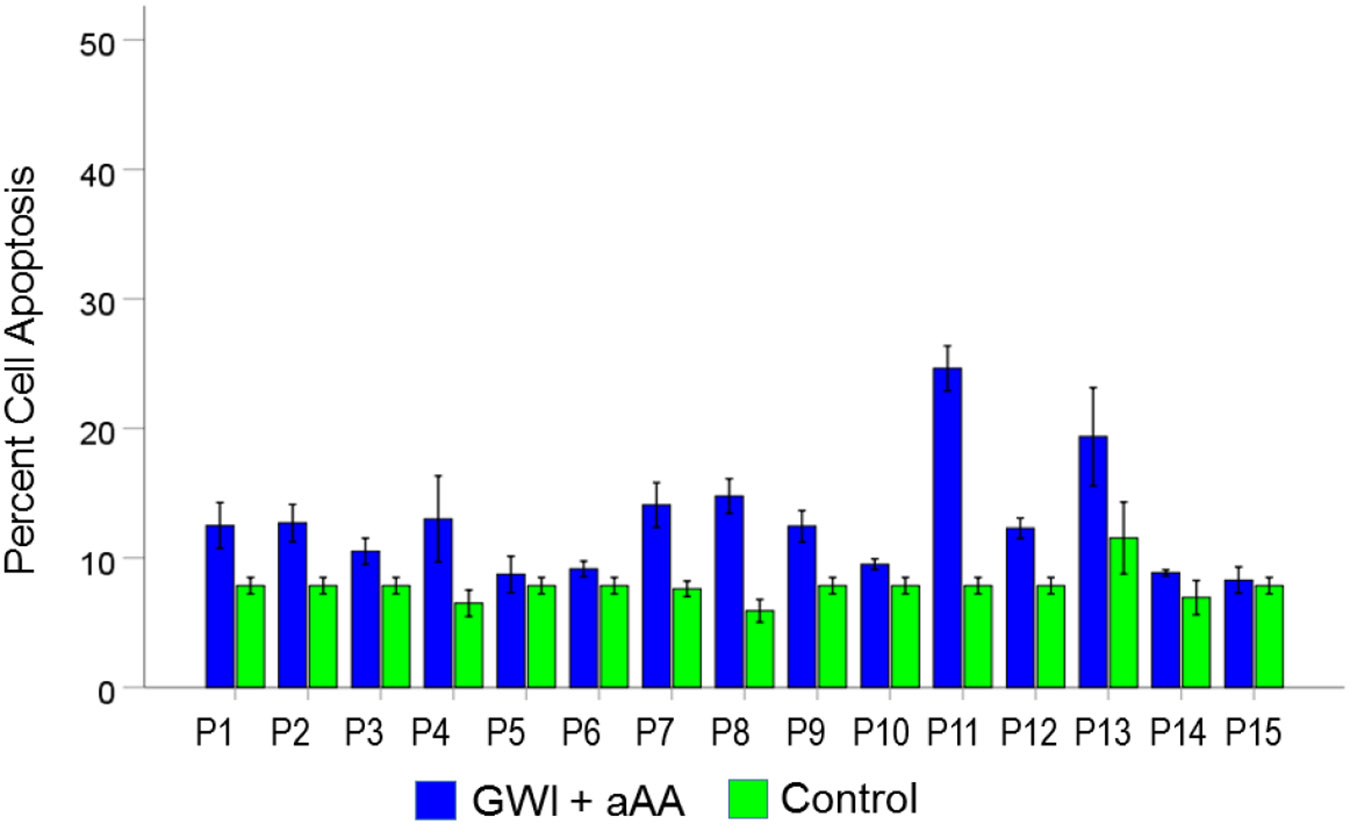
Mean (± SEM) percent cell apoptosis for each one of the 15 GWI patients (P1-P15, N = 5 per patient) in the conditions shown (GWI+aAA, Control). Notice that cell apoptosis in the GWI+aAA condition is close but systematically higher than that in Control. See text for details.

Figures 9, 10, and 11 show the effects of the 3 pair of treatments on cell spreading for each one of the 15 GWI patients (GWI vs. Control, GWI vs. GWI+aAA, and GWI+aAA vs Control, respectively). It can be seen that the general effects described above were observed in each patient. Similarly, Figures 12-14 show that the effects on cell apoptosis were consistently observed in each patient.

## Discussion

In the present study we investigated the effect of antianthrax antibodies on the detrimental effects of GWI serum regarding cell spreading and apoptosis in neuroblastoma cell cultures. We found that the addition of anti-anthrax antibodies to the GWI serum resulted in a substantial and highly significant increase in cell spreading and, concomitantly, a reduction in cell apoptosis, that is, an overall improvement in the cell culture. Remarkably, this beneficial effect was observed in each one of the 15 GWI patients studied, documenting the consistency of the effect. These results indicate, for the first time, the presence of harmful anthrax antigen in the serum of GWI patients and document the ameliorating effect of its removal by specific anti-anthrax antibodies. Given the rarity of exposure to anthrax and vaccination against anthrax in the general population, it is most likely that the anthrax antigen in the serum of GWI veterans is due to (a) their vaccination against anthrax, combined with (b) their inability to make antibodies to eliminate it. Indeed, the Gulf War was the first time that a large number of military personnel received the anthrax vaccine (“Biothrax”; https://www.rxlist.com/biothrax-drug.htm#indications and https://www.fda.gov/media/71954/download^22^). The vaccine component PA83 is an 83kDa precursor polypeptide consisting of 735 amino acids which binds to anthrax toxin receptors. There are two distinct toxin cell receptors, ANTXR1 (TEM8, Tumor endothelial marker 8) and ANTXR2 (CMG2, Capillary morphogenesis protein 2) which are widely expressed in cells. After binding to its receptor(s), PA83 is cleaved by cellular proteases of the furin family, or by serum proteases to generate a nicked 20 kDa fragment (PA20) at N-terminal and a 63 kDa fragment (PA63) at C-terminal. The 63 kDa fragment self-associates to form a prepore which is a heptameric ring and can bind other toxins of anthrax (edema factor: EF and lethal factor: LF). Eventually the prepore makes a translocate channel after inserting into the membrane. This channel is used for translocation of LF and EF into the cytoplasm by enzymatically disrupting the host cell^23^. Although PA was previously believed to only mediate entry of lethal factor or edema factor, it was found to be toxic to several cell types in culture, including Chinese hamster ovary (CHO) cells. In cultures of these cells which express the TEM receptor, PA63 made the plasma membrane permeable leading to apoptosis^24^. N2A cells can also bind the PA, since the TEM receptor contains an extracellular domain related to von Willebrand factor /integrin inserted domain^25,26^ and N2A cells express several integrin receptors^27,28^. For this binding to occur, persistence of PA antigen from the anthrax vaccine administered to GW veterans is a prerequisite. Indeed antigens have been described to persist for prolonged times following immunization in lymphatic endothelial cells^19^ and lymphoid follicles of specifically immunized mice^20^. The possibility then exists that persisting pathogens circulate in the blood stream and remain in the serum for an extended time, also affecting permeability of the blood-brain-barrier^29^. Compromised BBB permeability would allow access of the anthrax pathogen to the brain leading to neural cell dysfunction and eventually loss. Indeed, the observed subcortical brain atrophy in GWI^13^ would be compatible with pathogen-induced cell damage and loss.

Given that veterans suffering from GWI lack specific protective adaptive immunity^8^, we hypothesize that the persistence of PA anthrax antigen in GWI serum may be due to the inability to mount antibodies against it, a hypothesis that remains to be evaluated.

Finally, since antibodies to anthrax PA could substantially protect from abnormal neural function in vitro, additional studies are warranted to establish whether these in vitro observations may provide strategies for in vivo intervention with GWI. The specific target is anthrax persisting antigen(s) which could be neutralized (by administering specific antibodies against them) and/or removed (by plasmapheresis) as a means to alleviate symptoms of the disease. Given (a) that the availability of such antibodies is very restricted and set aside only for emergencies, and (b) that exposure or re-vaccination to anthrax of GW veterans is improbable, an alternative effective intervention could be plasmapheresis, a procedure by which plasma with harmful substances is replaced successively, so that such substances are eliminated from the circulation. This potentially effective intervention remains to be evaluated in clinical trials.

## Figures and Tables

**Figure 2.**
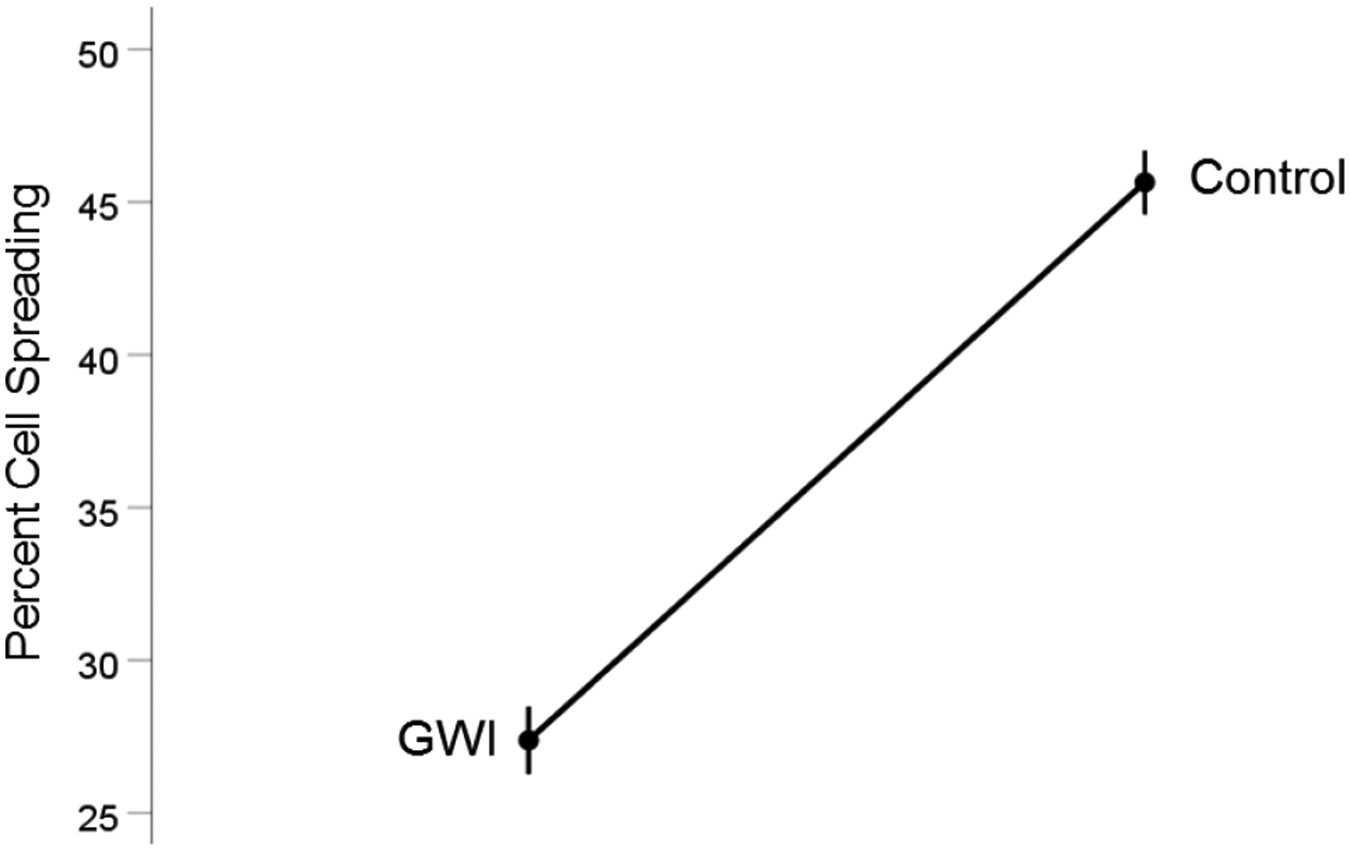
Mean (± SEM) percent cell spreading for the GWI and Control groups (N = 75 for each). Notice the reduction of cell spreading in GWI. See text for details.

**Figure 3.**
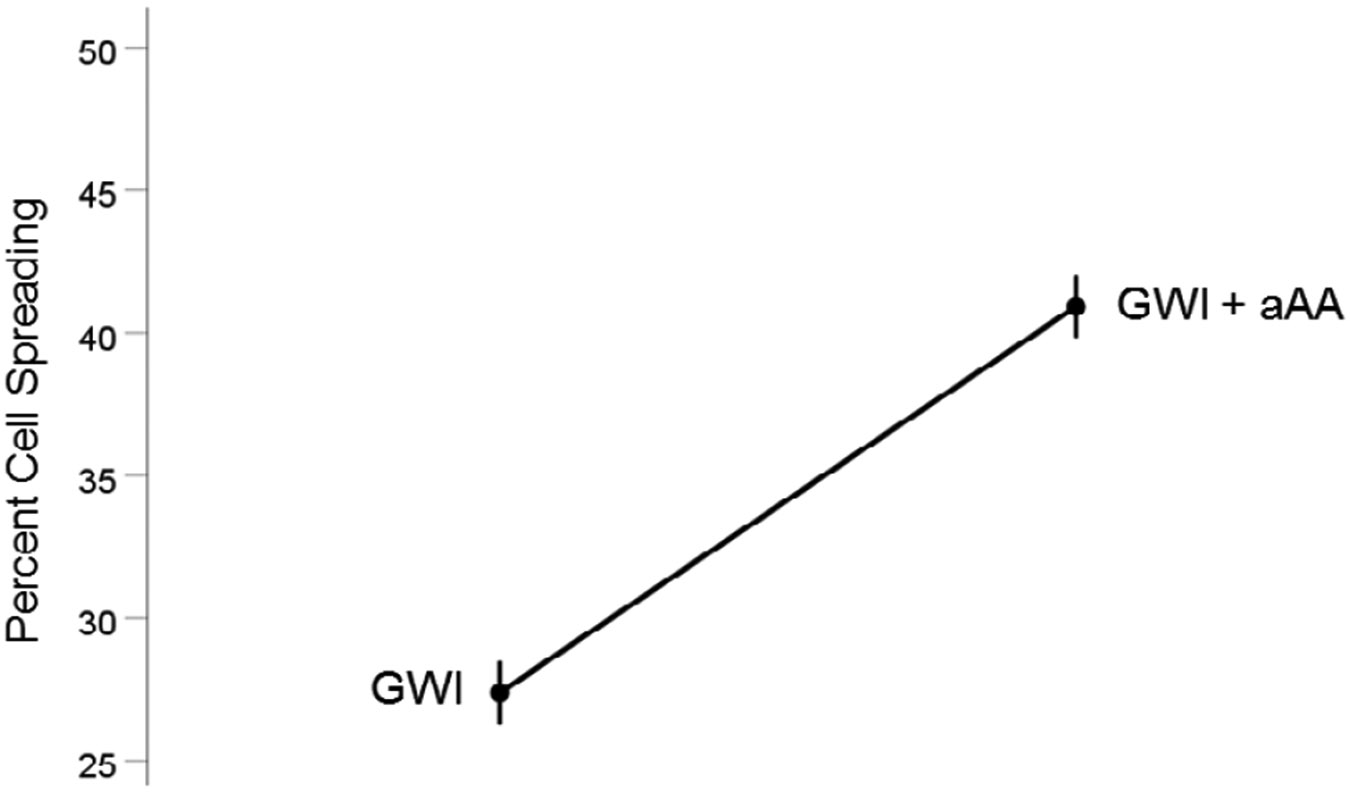
Mean (± SEM) percent cell spreading for the GWI and GWI+aAA groups (N = 75 for each). Notice the improvement of cell spreading in GWI+aAA. See text for details.

**Figure 4.**
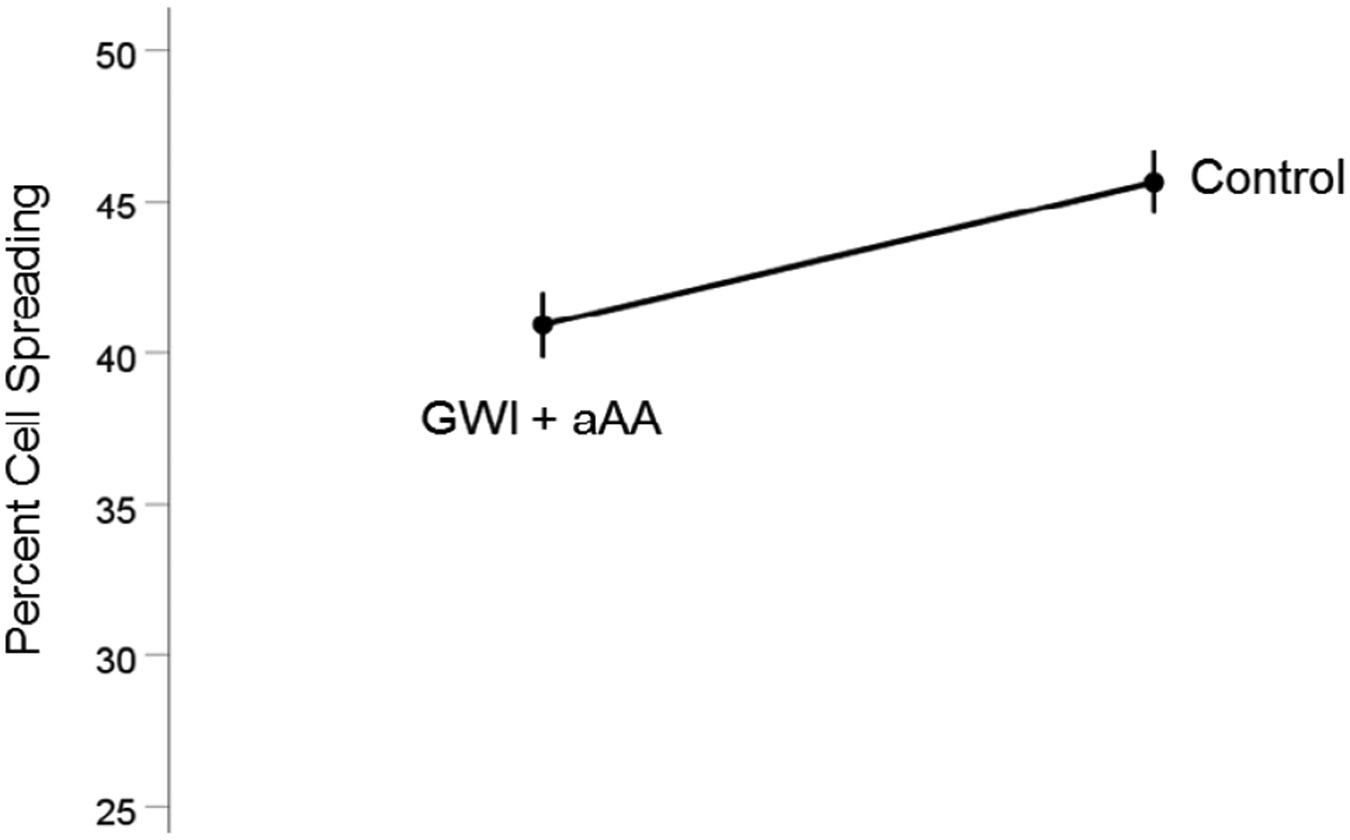
Mean (± SEM) percent cell spreading for the GWI+aAA and Control groups (N = 75 for each). Notice that cell spreading in GWI+aAA is close to, but lower than, the Control. See text for details.

**Figure 6.**
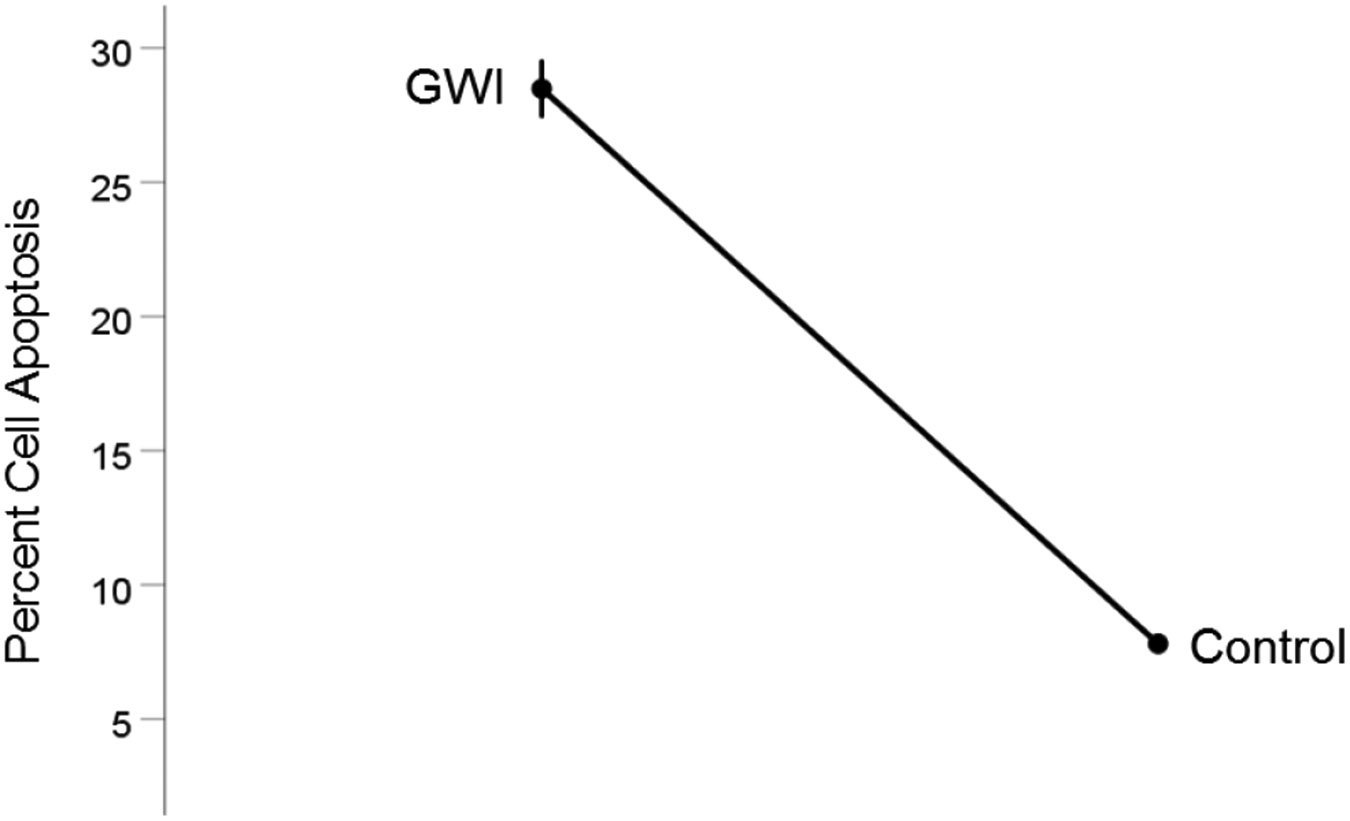
Mean (± SEM) percent cell apoptosis for the GWI and Control groups (N = 75 for each). Notice the increase in cell apoptosis in GWI. See text for details.

**Figure 7.**
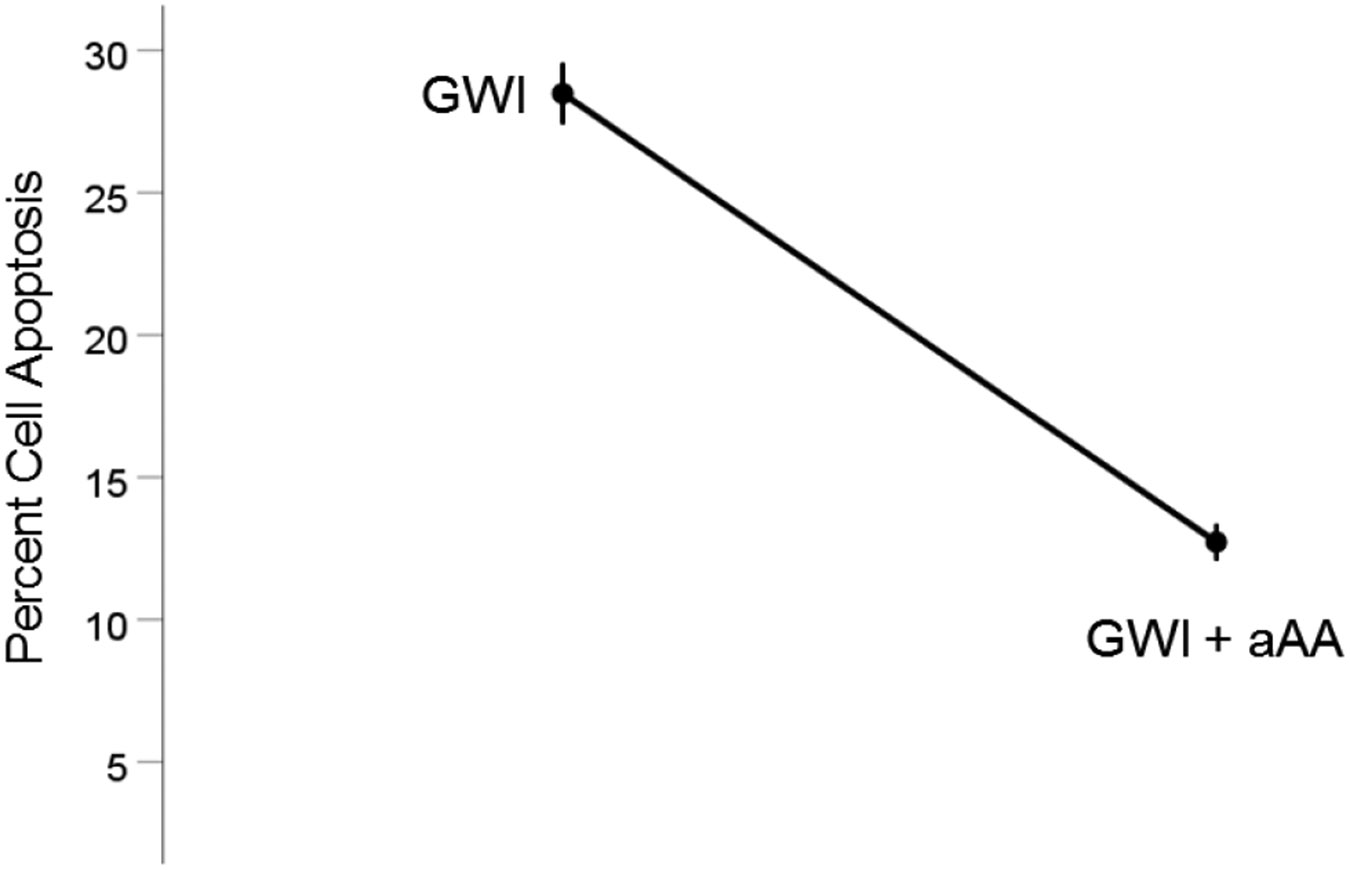
Mean (± SEM) percent cell apoptosis for the GWI and GWI+aAA groups (N = 75 for each). Notice the decrease in cell apoptosis in GWI+aAA. See text for details.

**Figure 8.**
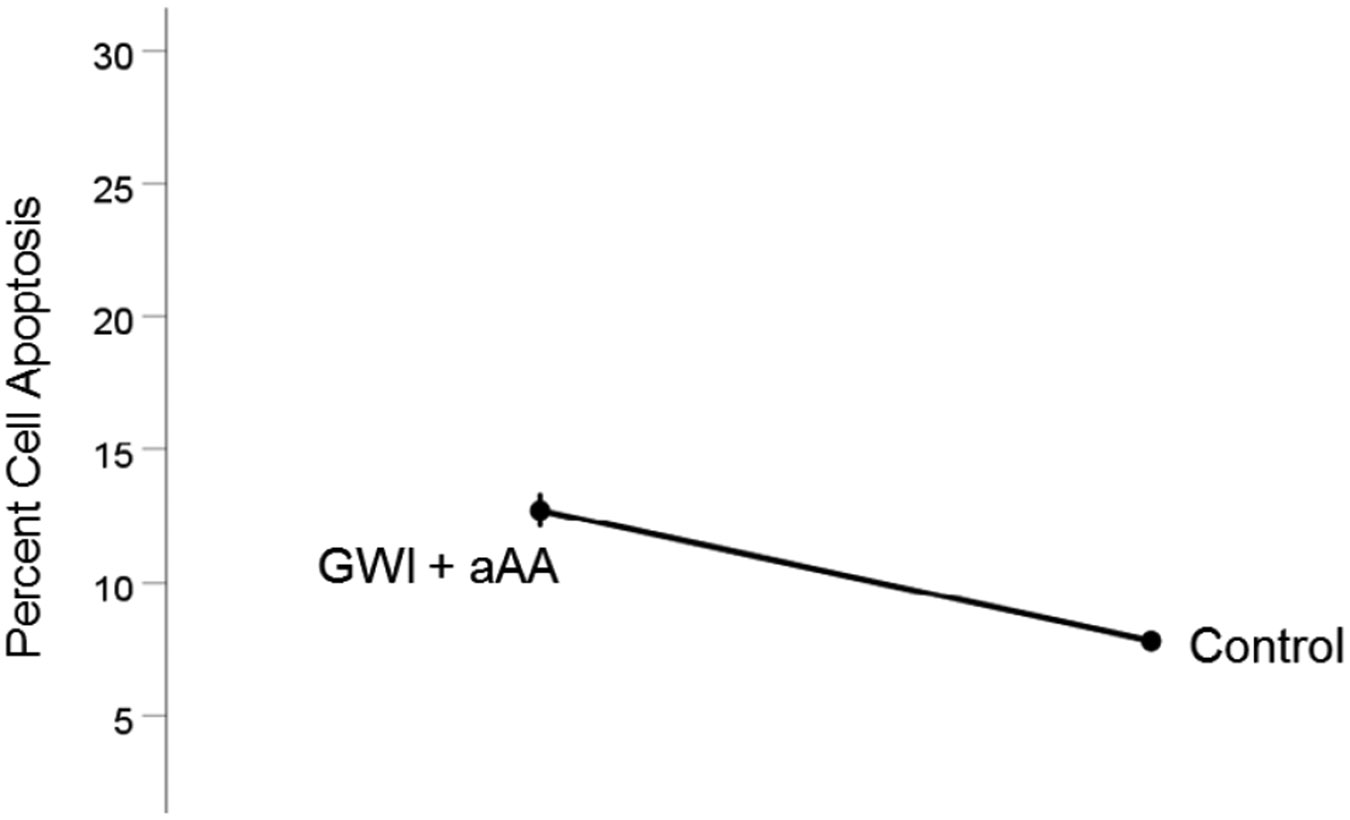
Mean (± SEM) percent cell apoptosis for the GWI+aAA and Control groups (N = 75 for each). Notice that cell apoptosis in GWI+aAA is close to, but higher than, the Control. See text for details.
